# Sales of Veterinary Antibiotics in Serbia: Identification of Problem Areas Using Standardized Metrics

**DOI:** 10.3390/ani14223201

**Published:** 2024-11-08

**Authors:** Ana Tomas, Nebojša Pavlović, Saša Vukmirović, Zorana Kovačević, Tihomir Dugandžija, Dragana Radovanović, Nebojša Stilinović

**Affiliations:** 1 Department of Pharmacology, Toxicology, and Clinical Pharmacology, Faculty of Medicine, University of Novi Sad, Hajduk Veljkova 3, 21000 Novi Sad, Serbia; ana.tomas@mf.uns.ac.rs (A.T.); sasa.vukmirovic@mf.uns.ac.rs (S.V.); 2 Department of Pharmacy, Faculty of Medicine, University of Novi Sad, Hajduk Veljkova 3, 21000 Novi Sad, Serbia; nebojsa.pavlovic@mf.uns.ac.rs; 3 Department of Veterinary Medicine, Faculty of Agriculture, University of Novi Sad, Trg Dositeja Obradovica 8, 21000 Novi Sad, Serbia; zorana.kovacevic@polj.edu.rs; 4 Department of Epidemiology, Faculty of Medicine, University of Novi Sad, Hajduk Veljkova 3, 21000 Novi Sad, Serbia; tihomir.dugandzija@mf.uns.ac.rs; 5 Oncology Institute of Vojvodina, Put Doktora Goldmana 4, 21204 Sremska Kamenica, Serbia; dragana.radovanovic@mf.uns.ac.rs; 6 Department of Anesthesia and Perioperative Medicine, Faculty of Medicine, University of Novi Sad, Hajduk Veljkova 3, 21000 Novi Sad, Serbia

**Keywords:** drug utilization research, population correction unit, veterinary antibiotics, phytobiotics

## Abstract

One Health, as a multi-disciplinary approach, considers animal, human, and environmental health. Serbia continuously records high rates of antimicrobial resistance, with a knowledge gap on practices regarding veterinary antibiotic use, as no standardized data regarding veterinary consumption of antibiotics that would allow for temporal or spatial comparison are available in Serbia. This study aimed to describe the sales of veterinary antibiotics in Serbia. Aggregated wholesale data on veterinary drug sales were retrieved from the annual reports available from the Medicines and Medical Devices Agency of the Republic of Serbia for a period between 2017 and 2020. Veterinary use was analyzed using standard European Medicines Agency proposed methodology and expressed in milligrams of active substance per population correction unit (mg/PCU). Overall, there has been a 13% increase in aggregated sales, in mg/PCU, of antibiotic veterinary medicinal products (VMPs) for food-producing animals in Serbia between 2017 and 2020. With sales of around 110 mg/PCU in 2020, Serbia would have ranked ninth in Europe. In 2020, the overall highest-selling antibiotic classes were penicillins (27.62 mg/PCU), tetracyclines (27.54 mg/PCU), and aminoglycosides (12.8 mg/PCU), accounting for 61.9% of the total sales of antibiotic VMPs for food-producing animals, in mg/PCU. An increase in the use of macrolides and lincosamides and a decrease in the use of penicillins and the group classified as “other antibacterials” was noted. The current study identified an increase in the sales of veterinary antibiotics in Serbia and pointed out several potential problem areas.

## Simple Summary

Antimicrobial resistance is a significant issue in Serbia, with limited data available on veterinary antibiotic usage. The results of the analysis of veterinary antibiotic sales in Serbia from 2017 to 2020 showed a 13% increase in antibiotic sales for food-producing animals. Based on our analysis, with sales of 110 mg/PCU in 2020, Serbia would have ranked ninth in Europe, well behind the goal set by the European Union (59.2 mg/PCU). Tetracyclines, penicillins, and aminoglycosides were the most sold antibiotics, making up 61.9% of total sales. An increase in the use of macrolides and lincosamides was noted. We need to take urgent action to curb antimicrobial resistance in Serbia, and the potential overuse and/or misuse of antibiotics in food-producing animals needs to be considered.

## 1. Introduction

Antibiotics are widely used drugs, not only in human medicine but also in veterinary medicine, agriculture, and aquaculture [1]. Antimicrobial resistance (AMR) development is driven by the overuse and/or misuse of antimicrobials, where under selective pressure, pathogens with resistant traits multiply and strive [2,3]. The issue is further exacerbated by improper waste management, leading to residues of antibiotics being identified in soil and groundwater, affecting the whole ecosystem [4,5,6].

For many years, reports on the problem of AMR and bacterial infections being increasingly difficult to treat have been available [1]. According to a landmark study published in *The Lancet* in 2019, around 1.27 million people died due to infections caused by antibiotic drug-resistant bacteria [7]. AMR is a critical global issue affecting human, environmental, and animal health, and with this being a complex problem, it is necessary to consider it interdisciplinary, and frame it within the One Health approach [8]. The World Health Organization (WHO) has established a formal alliance to enhance global coordination and promote intersectoral collaboration between the public health and animal health sectors as well as in food safety, with AMR being identified as one of the three priority topics for joint actions [3]. All sectors should combine forces to tackle the problem of AMR. One of the efforts aims to reduce and improve the quality of the use of antibiotics in animals, with different legislation in place. In Serbia, the regulation of veterinary antibiotics aligns with the EU standards, as the country is currently in the process of EU integration. The primary legislation includes the Law on Veterinary Matters and the Law on Medicines and Medical Devices [9,10]. Overall, the regulation provides detailed guidance on the usage of veterinary medicines, promoting the rational use of antibiotics only when clinically justified and emphasizing the prudent use of antibiotics important for human health, in line with the Serbian National AMR Action Plan [11].

An analysis of antibiotic utilization is the first and necessary step for the development of antibiotic stewardship programs needed in tackling AMR effectively. Since Serbia is among the countries that record some of the highest antibiotic use in human medicine in Europe [2], the use of veterinary antibiotics should also be considered. Currently, no standardized data regarding the veterinary use of antibiotics that would allow for temporal or spatial comparison are available and accessible in Serbia. The European Medicine Agency (EMA) publishes annual reports on veterinary antibiotic sales, but these do not include data for Serbia [12]. Therefore, this study aimed to describe the veterinary use of antibiotics in Serbia using standardized metrics.

## 2. Materials and Methods

### 2.1. Data Sources and Data Extraction

Information about the sales of veterinary antibiotics was retrieved from annual reports available from the Medicines and Medical Devices Agency of the Republic of Serbia (ALIMS) for a period between 2017 and 2020 [13,14]. These reports contain information on the sales of all veterinary drugs for the following years, organized by the Anatomical Therapeutic Chemical (ATC) classification codes [15], with information on the international non-proprietary name, dose and dosage form, and number of packages sold. The information is based on data reported by wholesalers in veterinary drug reports submitted to ALIMS for each respective year. Data on veterinary antibiotic use in other European countries were extracted from the interactive European Surveillance of Veterinary Antimicrobial Consumption (ESVAC) database [16].

Based on the ESVAC protocol, the following groups and ATCvet codes (ATC codes version 2021) were extracted for Serbia:

- Antimicrobial substances for intestinal use (QA07AA and QA07AB);
- Antimicrobial substances for intrauterine use (QG01AA, QG01AE, QG01BA, QG01BE, QG51AA, and QG51AG);
- Antimicrobial substances for systemic use (QJ01);
- Antimicrobial substances for intramammary use (QJ51);
- Antimicrobial substances used as antiprotozoals (QP51AG).

The substances were presented according to the classes/subclasses defined by the ATCvet hierarchical system using the WHO international non-proprietary names (INN).

### 2.2. Metrics

The main measurement unit was milligrams (mg) of active substance normalized by the population correction unit (PCU). Based on the number of packages sold, the pharmaceutical form, the strength of the antibiotic active substance(s), and the pack size, the quantity of antibiotic active substance in tonnes sold was calculated by multiplying the number of packages sold by the strength of the antibiotic active substance per package unit. We have used conversion factors to convert international units (IU) into mg when the strength was reported in IU or to calculate the mass of antibiotic active moiety in mg when the strength was reported as the derivative/compound strength. These conversion factors were based on the 2021 ESVAC sales data reporting form and protocol [17]. For fixed combinations containing multiple active substances, the quantity of each antibiotic active substance was calculated separately.

The denominator used to standardize the data was PCU, describing the estimated weight at the treatment of livestock and slaughtered animals in the respective year. Thus, PCU is a technical measurement unit, and in this study, 1 PCU corresponds to 1 kg of different categories of livestock and slaughtered animals. This measure has been established as a denominator for the sales data and serves to normalize the total quantities of antibiotic active substances sold for the animal population that could be potentially treated with these. The PCU only includes food-producing animals, including horses and farmed fish, as population data of companion animals, such as dogs and cats, and other animals potentially treated, such as pigeons, were not available. Therefore, tablets were excluded from the data sets prior to the normalization of sales by PCU since they are typically approved for companion animals only. The animal demographics in Serbia were obtained from the Statistical Yearbook by the Statistical Office of the Republic of Serbia for the years 2017 to 2020. The instruments used by the Statistical Office, along with their coverage, characteristics, and the standardization of concepts and definitions, adhered to the EUROSTAT methodology for farm structure surveys and agricultural production methods [18,19,20,21].

The main measurement unit (mg of active substance normalized by the population correction unit) was calculated as follows:

$$\frac{mg}{PCU}=\frac{Quantity\;sold\;in\;tonnes\times 10^{9}}{PCU\;in\;kg}$$

### 2.3. Data Analysis

Antibiotic sales in mg/PCU were analyzed based on ATC class and subclass, product form, and EMA classification system. Additionally, harmonized outcome indicators were used—the overall sales of veterinary antimicrobials and the proportion of sales of 3rd- and 4th-generation cephalosporins, quinolones, and polymyxins of total sales in mg/PCU.

Product form classification was based on the ESVAC definition of “product forms”, which combines pharmaceutical form and the route of administration. These included injectable products, intramammary products, oral solutions (including powders and concentrates for administration in drinking water), oral powders (powder to be administered with feed), and premixes. The EMA’s classification system served as the framework for categorizing antimicrobials into four groups for use in both food-producing and pet animals. Per this categorization, antibiotics are grouped in category A (“Avoid”) antibiotics that cannot be used in food-producing animals and should only be administered to individual companion animals in exceptional cases. Category B (“Restrict”) antibiotics are of critical importance in human medicine, and their use in animals should be limited to reduce the risk to public health. Category C (“Caution”) antibiotics should be used only when no antimicrobial agents from category D would be clinically effective. Category D (“Prudence”) includes antibiotics that should be used as first-line treatments whenever possible [22,23].

The results were analyzed in the Microsoft Excel 2019 software and presented as frequencies and absolute numbers. The relative percent change was calculated as follows: (value in 2020—value in 2017)/value in 2017*100. The results were presented as tables and graphs.

## 3. Results

**Figure 1:**
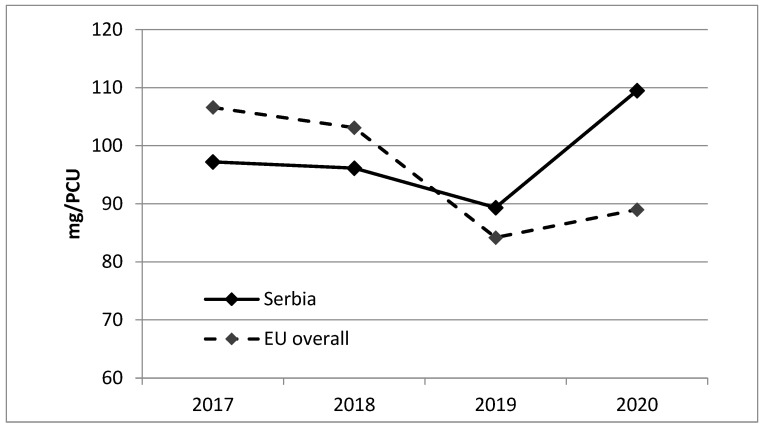
Aggregated sales, in mg/PCU, of antibiotic VMPs for food-producing animals in Serbia vs. EU overall (2017–2020).

Figure 1 compares the aggregated sales of antibiotic veterinary medicinal products for food-producing animals in Serbia and the EU overall, measured in mg/PCU for the years 2017 to 2020. Serbia’s antibiotic sales fluctuated, with a decrease from 2017 to 2019, followed by a notable rise in 2020, having total sales of around 110 mg/PCU. The EU overall saw a consistent decrease in antibiotic sales until 2020 (89 mg/PCU), when it slightly increased compared to 2019 (84.2 mg/PCU). Serbia’s antibiotic sales surpassed the EU average in 2020, whereas in previous years, Serbia’s levels were generally lower or similar to the EU’s.

**Figure 2:**
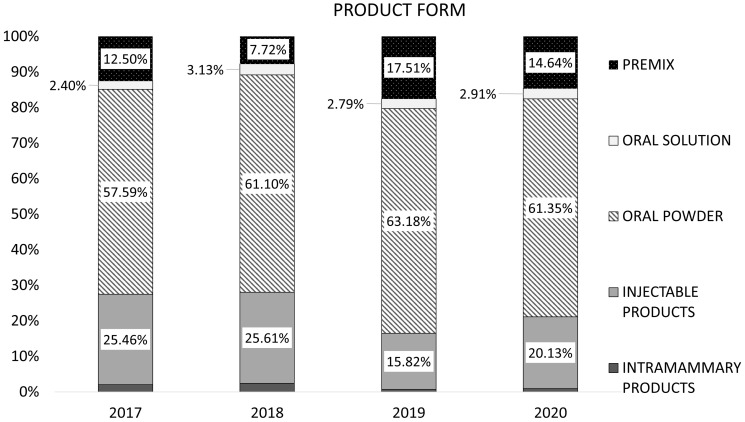
Proportion of aggregated sales, in mg/PCU, of antibiotic VMPs for food-producing animals by product form in Serbia (2017–2020).

The aggregated sales of antibiotic VMPs for food-producing animals, stratified by product form, are shown in Figure 2. Oral powders were the highest-selling product form, accounting for 61.4% of the total sales (mg/PCU) of antibiotic VMPs, followed by injectable products (20.1%) and premixes (14.6%).

As shown in Table 1, in 2020, the overall highest-selling antibiotic classes were penicillins (27.62 mg/PCU), tetracyclines (27.54 mg/PCU), and aminoglycosides (12.8 mg/PCU), accounting for 61.9% of the total sales of antibiotic VMPs for food-producing animals, in mg/PCU. An increase in the use of macrolides and lincosamides and a decrease in the use of other antibacterials (other antibacterials included spectinomycin, bacitracin, and novobiocin) was noted. Overall, there has been a 13% increase in aggregated sales, in mg/PCU, of antibiotic VMPs for food-producing animals in Serbia between 2017 and 2020.

As shown in Table 2, in 2020, the changes observed were most pronounced for macrolides, with a 134% increase, but the increase in the use of beta-lactamase-resistant penicillins, trimethoprim, and 3rd-generation cephalosporins was also observed.

From 2017 to 2020, veterinary antibiotic sales in Serbia showed no use of A category antibiotics, while the majority consistently fell under the D category, with a gradual increase in C category antibiotics and fluctuating levels in the B category (Table 3).

**Figure 3:**
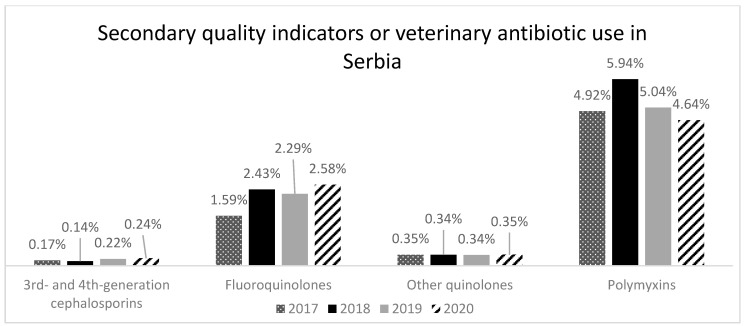
Proportion of sales of 3rd- and 4th-generation cephalosporins, fluoroquinolones, other quinolones, and polymyxins of total sales, in mg/PCU, of antibiotic VMPs for food-producing animals in Serbia from 2017 to 2020.

The secondary outcome indicators of antibiotic consumption correspond to the total sales (mg/PCU) of those antibiotic VMPs for food-producing animals that are classified as highest priority critically important antimicrobials by the WHO: 3rd- and 4th-generation cephalosporins, quinolones (indicating the proportion of fluoroquinolones), and polymyxins. The proportion of total aggregated sales corresponding to each of these antibiotic classes varied between the 4 years, ranging from 0.14% to 0.24% for 3rd- and 4th-generation cephalosporins, 1.59% to 2.58% for fluoroquinolones, and 4.64% to 5.94% for polymyxins (Figure 3). Sales trends, in mg/PCU, for other quinolones were stable.

## 4. Discussion

There are gaps in knowledge on veterinary antibiotic use in Serbia, making efforts to tackle AMR while considering the One-health perspective difficult. The present study aimed to contribute to closing this knowledge gap by providing standardized data that can be used for benchmarking purposes and policy development.

Based on the results of the present study, with sales of around 110 mg/PCU, Serbia would have ranked ninth in Europe in 2020, considering the wide range of reported sales, with Cyprus ranking first (344 mg/PCU) and Norway reporting the lowest use (2 mg/PCU). In 2020, Serbia had lower sales than some of the neighboring countries, Bulgaria (121 mg/PCU) and Hungary (163 mg/PCU). In the following years, the use decreased, resulting in Bulgaria and Hungary reporting sales of 103.2 mg/PCU and 111 mg/PCU in 2022, respectively [12]. According to the Farm to Fork Strategy, the target of a “50% reduction in EU overall sales of antimicrobials for farmed animals and in aquaculture” was set at 59.2 mg/PCU for 2030 based on EU reference values in 2018, putting Serbia well behind this goal [24]. Efforts made to optimize antibiotic use in animals were successful in many countries, and most EU states are already well below the goal set for 2030.

Overall, there has been a 13% increase in the aggregated sales of antibiotic VMPs for food-producing animals in Serbia between 2017 and 2020. The latest ESVAC report calculated the total aggregated sales of 84.8 mg/PCU for 2022 across all 31 reporting countries [11,12]. This represents a 12.7% overall decline compared to 2021 (a reduction of 10.7 mg/PCU). A large difference continues to be observed between countries with the highest and lowest sales, ranging from 2.1 mg/PCU to 254.6 mg/PCU. However, as it is stated in the thirteenth ESVAC report, the variation between countries can be partly attributed to differences in animal demographics, disease prevalence, production systems, prescription practices, daily doses of antimicrobial agents, pharmaceutical forms, and the duration of treatment. ESVAC emphasizes the fact that country-specific factors should be considered when evaluating results on a country-by-country basis, avoiding direct comparisons [12]. The exact reason for the observed increase in Serbia is difficult to pinpoint, but as this year covers the period during the Coronavirus Disease 2019 pandemic, which influenced society as a whole, some possible explanations include changes in the prescribing behavior of veterinarians, changes in the enforcement of regulations on antibiotic use during the pandemic, and issues with access to alternatives such as vaccines. Another possible explanation is more accurate data collection and reporting practices.

In Serbia, oral powders were the highest-selling product form, followed by injectable products and premixes. The overall sales figures for oral powders, in addition to the sales of premixes, provide a reasonable estimate for group treatment, including groups in one pen/farm. These results are comparable to those obtained in other countries, where in 2021, 86.3% of total sales of antibiotic VMPs for use in food-producing animals were of VMPs predominantly used for group treatment [12]. Antibiotics are mainly administered by three different routes: by injection, via medicated feed, or via drinking water. After the administration of antibiotics, between 30% and 90% of the initial dose given is excreted as active metabolites or as non-metabolized form. Therefore, high concentrations of antibiotics and/or their metabolites can be present in urine or feces [25,26]. Manure is used as land fertilizer for its high levels of phosphorus, nitrogen, and organic matter that can improve the physical and chemical properties of soil and provide nutrients to plants. The application of manure as fertilizer is a common practice in many countries, and residues of antibiotics excreted and present in the animal manures/feces enter the environment either by spreading livestock waste onto agricultural fields as fertilizer or in the form of sludge after manure collection and storage. Antibiotics present in manures/feces can be a risk for humans and the environment [26].

In Serbia, in 2020, the overall highest-selling antibiotic classes were penicillins (27.62 mg/PCU), tetracyclines (27.54 mg/PCU), and aminoglycosides (12.8 mg/PCU), accounting for 61.9% of the total sales of antibiotics. In Europe, in 2021, the overall highest-selling antibiotic classes were penicillins (31.2%), tetracyclines (25.8%), and sulfonamides (9.9%), accounting for 66.9% of the total sales of antibiotic VMPs. In the Nordic countries and Switzerland, where the sales of penicillins are typically high, beta-lactamase-sensitive penicillins were the highest-selling penicillin subclass (representing between 62% and 96% of total penicillin). For the remaining countries, penicillins with extended spectrum (98.0% amoxicillin, 2.0% ampicillin, and <0.01% metampicillin) accounted for the main proportion of penicillin sales [12]. The EMA introduced a classification of veterinary antibiotics, aiming to help limit the use of antibiotics most detrimental to the development of AMR, considering potential public health consequences and the need for their use in veterinary medicine. A positive finding is that category A antibiotics, which are not to be used in food-producing animals, were completely absent from veterinary antibiotic sales in Serbia from 2017 to 2020. Additionally, category B antibiotics, critical for human medicine, remained consistently below 11%, aligning with responsible use principles. Most antibiotics sold during this period belonged to category D, which are recommended as first-line treatments but should still be used prudently and only when medically necessary. However, the proportion of category D antibiotics showed a slight decrease over time, while the share of category C antibiotics increased, suggesting a potential trend toward the use of higher-risk antibiotics that requires careful attention [22,23].

In Serbia, an increase in the use of macrolides and lincosamides and a decrease in the use of other antibacterials (which included spectinomycin, bacitracin, and novobiocin) was noted. The use of fluoroquinolones was also marked. In Serbia, in human medicine, macrolides, fluoroquinolones, and broad-spectrum cephalosporins are commonly used [2]. These classes require special considerations, as by the WHO, they are classified as Watch-group antibiotics, which have a higher resistance potential when compared to the Access-group antibiotics [2]. A recent report prepared by the European Centre for Disease Prevention and Control (ECDC), European Food Safety Authority (EFSA), and EMA on the integrated analysis of the possible relationship between antimicrobial consumption in human and veterinary medicine and the occurrence of AMR in bacteria from humans and food-producing animals found several associations [27]. For example, a statistically significant positive association between the consumption of fluoroquinolones and macrolides in humans and the consumption of these antimicrobials in food-producing animals was found, with countries with high consumption in humans also tending to have high consumption in food-producing animals and vice versa, which is in line with our findings. What is more worrisome, this report found a statistically significant positive association between the consumption of fluoroquinolones, polymyxins, and tetracyclines in humans and food-producing animals, and resistance in indicator *E. coli* [27]. In Serbia, based on the Central Asian and Eastern European Surveillance of Antimicrobial Resistance (CAESAR) data, between 2017 and 2021, resistance levels in *P. aeruginosa* and *E. coli* to quinolones increased from 56.7% to 67.6% and 40.4% to 45.3%, respectively. For *K. pneumoniae*, high levels of resistance, including carbapenems (62.7%), almost 90% to quinolones, and 91.9% to third-generation cephalosporins, were present in 2021. In 2021, 53.3% of all *P. aeruginosa* and 71.7% of all *K. pneumonia* isolates in Serbia were multidrug-resistant. High levels of resistance were also reported for penicillin (47.6%) and macrolides (27.3%) in *S. pneumoniae* [28]. In the United States, Canada, and European Union countries, AMR in animals has been the focus of systematic surveillance for decades [29]. Data on AMR in animals are scarcer for Serbia, as no comprehensive surveillance and monitoring program for AMR in zoonotic and indicator bacteria is implemented at the moment, contributing to the knowledge gap. A study on AMR of commensal *E. coli* of food-producing animals from three farms in the northern part of Serbia reported a very high prevalence of resistance to tetracyclines, and moderate levels to streptomycin, ampicillin, cephalothin, and nalidixic acid [30]. Multidrug resistance (MDR) was observed among all swine *E. coli* isolates, 63.2% of broiler isolates, and 37.5% of cattle isolates [30]. An analysis of AMR in Serbian poultry farms found resistance to erythromycin, streptomycin, fluoroquinolones, and tetracyclines, with 35% of the isolates having MDR [31].

In many countries, measures implemented to reduce antibiotic use in food-producing animals have been effective, but they need to be continuously reinforced. In addition to the public health impact, AMR negatively impacts livestock production and the economy, and without action, this will intensify over time. Estimates are that by 2050, the annual livestock production losses due to AMR will equal the consumption needs of 746 million people [32]. Effective antibiotic stewardship in agriculture has been shown to reduce resistance without harming farm productivity and profitability. Yet, obstacles in monitoring and regulatory control continue to hinder efforts to curb the excessive use of antibiotics in food production [33]. Conversely, in low-income and middle-income countries, the inappropriate use of antimicrobials is driven by weak regulatory policies, lack of veterinary guidelines, self-prescribing by farmers, and insufficient investment in animal health services compared to human health [34].

One of the priorities in overcoming challenges in antibiotic use in animals in our setting should be the establishment of regular surveillance and monitoring procedures, strict control of the implementation of preventative measures such as vaccination and hygiene practices, but also increasing prescriber competencies [35]. A recent study assessing farm animal veterinarians’ knowledge and attitudes toward antimicrobial use and AMR in Serbia found that although the majority of surveyed veterinarians agreed that AMR represents a severe concern in the health sector, more than two-thirds did not have adequate knowledge about antimicrobial stewardship, highlighting the need for increased education as a key priority in Serbia [36]. In addition to optimizing prescribing practices, another approach could be redirection to alternatives to antibiotics, such as organic acids, probiotics, and phytogenics [33]. A recent review highlighted the fact that various substances have shown potential as effective substitutes for antibiotics. Replacing antibiotics with these alternatives is crucial for reducing antibiotic contamination [37]. An example of such a practice is the project funded by the Dutch government for the creation of freely available materials to provide farmers and veterinarians with accessible information on natural health alternatives for livestock, including medicinal plants, initially targeting organic farmers but later expanding to conventional farmers to help reduce antibiotic use [38]. Such an approach certainly had an effect, as by 2022, with a score of 37.0 mg/PCU, the Netherlands far surpassed the EU target, especially when compared to the result of 146.0 mg/PCU for the year 2010, the year when the campaign started [12,38]. Finally, pharmacoeconomic analysis has shown that plant-based treatments can be considered as a cost-saving alternative to conventional antibiotics [39].

### Limitations

Due to the aggregated sales data used, it is not possible to identify the exact animal species and indications for which antibiotics were used. The data presented here should not be used for direct comparison with other countries, given that the findings may be influenced by factors such as type of data, animal demographics, types of animal production systems, disease incidence, or outbreaks, among others. Moreover, some of the sales allocated to food-producing animals could have been used for non-food-producing animals.

## 5. Conclusions

The study described the veterinary sales of antibiotics in Serbia and pointed out several potential problem areas, including an increase in overall use, an increase in the share of quinolones, and the high use of category C antibiotics, which should be considered only when there are no antibiotics in category D that could be clinically effective. Further studies, including detailed analyses of the practices of veterinarians in Serbia and the habits of farmers and livestock producers, are needed to fully grasp the problem. Cross-sectoral collaboration and actions to combat AMR are urgently needed in our setting.

## Figures and Tables

**Table 1:** Aggregated sales, in mg/PCU, of antibiotic VMPs for food-producing animals by antibiotic class in Serbia (2017–2020).

Class	Year				
	2017	2018	2019	2020	Relative Change 2017–2020
	mg/PCU	mg/PCU	mg/PCU	mg/PCU	%
Aminoglycosides	10.59	11.47	8.77	12.84	+21
Amphenicols	3.45	3.26	3.71	5.38	+56
Cephalosporins	0.27	0.25	0.29	0.38	+40
Macrolides and lincosamides	4.27	4.15	7.17	8.88	+108
Other antibacterials *	4.13	10.79	2.81	2.53	−39
Penicillins	30.47	27.32	16.58	27.62	−9
Pleuromutilins	4.90	4.46	6.17	5.06	+3
Polymyxins	4.78	5.71	4.50	5.08	+6
Quinolones	1.88	2.66	2.35	2.82	+50
Sulfonamides and trimethoprim	9.68	11.08	10.08	11.36	+17
Tetracyclines	22.78	14.99	26.92	27.54	+21
Total	97.21	96.15	89.35	109.49	+13

* other antibacterials included spectinomycin, bacitracin, and novobiocin.

**Table 2:** Aggregated sales, in mg/PCU, of antibiotic VMPs for food-producing animals by antibiotic subclass in Serbia (2017–2020).

Subclass	Year				Relative Change 2017–2020
	2017	2018	2019	2020	
	mg/PCU	mg/PCU	mg/PCU	mg/PCU	%
1st-generation cephalosporins	0.11	0.12	0.10	0.12	+9
3rd-generation cephalosporins	0.14	0.11	0.17	0.23	+64
4th-generation cephalosporins	0.02	0.02	0.02	0.03	+50
Aminoglycosides	10.59	11.47	8.77	12.84	+21
Amphenicols	3.45	3.26	3.71	5.38	+56
Beta-lactamase-resistant penicillins	0.10	0.15	0.15	0.22	+120
Beta-lactamase-sensitive penicillins	17.26	10.70	1.94	9.40	−46
Penicillins with extended spectrum	13.12	16.47	14.50	17.99	+37
Fluoroquinolones	1.54	2.33	2.04	2.82	+58
Lincosamides	0.69	0.86	0.56	0.50	−28
Macrolides	3.58	3.29	6.61	8.38	+134
Other antibacterials *	4.13	10.79	2.81	2.53	−39
Other quinolones	0.34	0.33	0.30	0.38	+12
Pleuromutilins	4.90	4.46	6.17	5.06	+3
Polymyxins	4.78	5.71	4.50	5.08	+6
Sulfonamides	9.13	10.41	9.33	10.41	+14
Tetracyclines	22.78	14.99	26.92	27.54	+21
Trimethoprim	0.55	0.67	0.75	0.95	+72
Total	97.21	96.15	89.35	109.49	+13

* other antibacterials included spectinomycin, bacitracin, and novobiocin.

**Table 3:** Use of veterinary antibiotics in Serbia based on categorization of antibiotics for use in animals for prudent and responsible use developed by EMA.

Year	Category			
	(A) AVOID (%)	(B) RESTRICT (%)	(C) CAUTION (%)	(D) PRUDENCE (%)
2017	0.00	7.96	24.58	67.46
2018	0.00	10.84	26.50	62.66
2019	0.00	8.86	31.41	59.73
2020	0.00	8.38	29.96	61.66

## Data Availability

All data are available upon reasonable request from the authors.
