# Correction: Tomas et al. Sales of Veterinary Antibiotics in Serbia: Identification of Problem Areas Using Standardized Metrics. *Animals* 2024, *14*, 3201

**DOI:** 10.3390/ani15203051

**Published:** 2025-10-21

**Authors:** Ana Tomas, Nebojša Pavlović, Saša Vukmirović, Zorana Kovačević, Tihomir Dugandžija, Dragana Radovanović, Nebojša Stilinović

**Affiliations:** 1 Department of Pharmacology, Toxicology, and Clinical Pharmacology, Faculty of Medicine, University of Novi Sad, Hajduk Veljkova 3, 21000 Novi Sad, Serbia; ana.tomas@mf.uns.ac.rs (A.T.);; 2 Department of Pharmacy, Faculty of Medicine, University of Novi Sad, Hajduk Veljkova 3, 21000 Novi Sad, Serbia; nebojsa.pavlovic@mf.uns.ac.rs; 3 Department of Veterinary Medicine, Faculty of Agriculture, University of Novi Sad, Trg Dositeja Obradovica 8, 21000 Novi Sad, Serbia; 4 Department of Epidemiology, Faculty of Medicine, University of Novi Sad, Hajduk Veljkova 3, 21000 Novi Sad, Serbia; 5 Oncology Institute of Vojvodina, Put Doktora Goldmana 4, 21204 Sremska Kamenica, Serbia; 6 Department of Anesthesia and Perioperative Medicine, Faculty of Medicine, University of Novi Sad, Hajduk Veljkova 3, 21000 Novi Sad, Serbia

## Table Legend

In the original publication [1], there was a mistake in the legend for Tables 1 and 2. Metronidazole was inadvertently mentioned. The correct legend appears below.

“* other antibacterials included spectinomycin, bacitracin, and novobiocin.”

## Error in Table

In the original publication [1], there were mistakes in Table 3 as published. The discrepancies arose from database structural issues, leading to errors across categories, including the mistaken inclusion of category A and inaccurate proportions of categories B, C, and D. A detailed manual review of the database was conducted to produce an accurate and meaningful representation of veterinary antibiotic use in Serbia. The corrected Table 3 appears below.

## Text Correction

There were errors in the original publication [1]. Metronidazole was mentioned in the text by mistake. To ensure clarity and accurately reflect the actual antimicrobial use and regulatory status, metronidazole was removed from the related text. A correction has been made to the Section 3 Results, Paragraph 3, second sentence: now it is “An increase in the use of macrolides and lincosamides and a decrease in the use of other antibacterials (other antibacterials included spectinomycin, bacitracin, and novobiocin) was noted.” and to the Section 4 Discussion, Paragraph 6, first sentence: now it is “In Serbia, an increase in the use of macrolides and lincosamides and a decrease in the use of other antibacterials (which included spectinomycin, bacitracin, and novobiocin) was noted.”

Another error was made in the original publication [1]. The paragraph in the Results section reflected the outdated results of the previous version of Table 3.

Based on the results presented in the updated Table 3, the paragraph has been revised accordingly. A correction has been made to the Section 3 Results, Paragraph 5: now it is “From 2017 to 2020, veterinary antibiotic sales in Serbia showed no use of A category antibiotics, while the majority consistently fell under the D category, with a gradual increase in C category antibiotics and fluctuating levels in the B category (Table 3).”

Additionally, part of the paragraph in the Section 4 Discussion discussed the outdated results of the previous version of Table 3.

Based on the results presented in the updated Table 3, the paragraph has been revised accordingly. A correction has been made to the Section 4 Discussion, Paragraph 5: instead of “A positive finding is that the use of category A antibiotics, which are not recommended for food-producing animals, has significantly decreased between 2017 and 2020, and the use of group B antibiotics of critical importance in human medicine has been below 10% in all the years studied. However, based on this categorization, the most used veterinary antibiotics in Serbia belong to category C [22]. Category C antibiotics should be used only when none of the category D antibiotics, recommended as first-line treatments, would be clinically effective. Needless to say, category D antibiotics should also be used prudently, only when medically needed [21,22]”, the corrected paragraph was inserted: “A positive finding is that category A antibiotics, which are not to be used in food-producing animals, were completely absent from veterinary antibiotic sales in Serbia from 2017 to 2020. Additionally, category B antibiotics, critical for human medicine, remained consistently below 11%, aligning with responsible use principles. Most antibiotics sold during this period belonged to category D, which are recommended as first-line treatments but should still be used prudently and only when medically necessary. However, the proportion of category D antibiotics showed a slight decrease over time, while the share of category C antibiotics increased, suggesting a potential trend toward the use of higher-risk antibiotics that requires careful attention [22,23].”

The authors apologize for any inconvenience caused and state that the scientific conclusions are unaffected. This correction was approved by the Academic Editor. The original publication has also been updated.

## Figures and Tables

**Table 3:** Use of veterinary antibiotics in Serbia based on categorization of antibiotics for use in animals for prudent and responsible use developed by EMA.

Year	Category			
	(A) AVOID (%)	(B) RESTRICT (%)	(C) CAUTION (%)	(D) PRUDENCE (%)
2017	0.00	7.96	24.58	67.46
2018	0.00	10.84	26.50	62.66
2019	0.00	8.86	31.41	59.73
2020	0.00	8.38	29.96	61.66
